# Organizational topology of brain and its relationship to ADHD in adolescents with d‐transposition of the great arteries

**DOI:** 10.1002/brb3.504

**Published:** 2016-06-09

**Authors:** Vincent J. Schmithorst, Ashok Panigrahy, J. William Gaynor, Christopher G. Watson, Vince Lee, David C. Bellinger, Michael J. Rivkin, Jane W. Newburger

**Affiliations:** ^1^ Department of Pediatric RadiologyChildrens Hospital of Pittsburgh of UPMCPittsburghPennsylvania; ^2^ Department of Radiology and BioinformaticsUniversity of PittsburghPittsburghPennsylvania; ^3^ Department of RadiologyChildren's Hospital Los AngelesLos AngelesCalifornia; ^4^ Brain and Creativity InstituteUniversity of Southern CaliforniaLos AngelesCalifornia; ^5^ Department of Cardiothoracic SurgeryChildren's Hospital of PhiladelphiaPhiladelphiaPennsylvania; ^6^ Department of NeurologyBoston Children's HospitalBostonMassachusetts; ^7^ Graduate Program for NeuroscienceBoston UniversityBostonMassachusetts; ^8^ Department of PsychiatryBoston Children's HospitalBostonMassachusetts; ^9^ Department of RadiologyBoston Children's HospitalBostonMassachusetts; ^10^ Department of NeurologyHarvard Medical SchoolBostonMassachusetts; ^11^ Department of CardiologyBoston Children's HospitalBostonMassachusetts; ^12^ Department of PediatricsHarvard Medical SchoolBostonMassachusetts

**Keywords:** Congenital heart disease, d‐TGA, fractional anisotropy, graph theory, MRI, neurodevelopment, tractography

## Abstract

**Objective:**

Little is currently known about the impact of congenital heart disease (CHD) on the organization of large‐scale brain networks in relation to neurobehavioral outcome. We investigated whether CHD might impact ADHD symptoms via changes in brain structural network topology in a cohort of adolescents with d‐transposition of the great arteries (d‐TGA) repaired with the arterial switch operation in early infancy and referent subjects. We also explored whether these effects might be modified by apolipoprotein E (*APOE*) genotype, as the *APOE* ε2 allele has been associated with worse neurodevelopmental outcomes after repair of d‐TGA in infancy.

**Methods:**

We applied graph analysis techniques to diffusion tensor imaging (DTI) data obtained from 47 d‐TGA adolescents and 29 healthy referents to construct measures of structural topology at the global and regional levels. We developed statistical mediation models revealing the respective contributions of d‐TGA, *APOE* genotype, and structural network topology on ADHD outcome as measured by the Connors ADHD/DSM‐IV Scales (CADS).

**Results:**

Changes in overall network connectivity, integration, and segregation mediated worse ADHD outcomes in d‐TGA patients compared to healthy referents; these changes were predominantly in the left and right intrahemispheric regional subnetworks. Exploratory analysis revealed that network topology also mediated detrimental effects of the *APOE* ε4 allele but improved neurobehavioral outcomes for the *APOE* ε2 allele.

**Conclusion:**

Our results suggest that disruption of organization of large‐scale networks may contribute to neurobehavioral dysfunction in adolescents with CHD and that this effect may interact with *APOE* genotype.

## Introduction

Congenital heart disease (CHD) is very common and affects 8 per 1000 live births (van der Linde et al. 2011). Approximately one‐third of affected children require intervention in early infancy. Improved survival has revealed that neurodevelopmental disability is the most common long‐term complication of CHD. Neurocognitive deficits have been documented in executive function, attention, visual‐spatial processing, and memory (Bellinger et al. 2011; Rollins et al. 2014). Structural brain abnormalities beginning in utero and extending into the postoperative period have also been shown, including reduced brain volume and increased risk of white matter injury (Limperopoulos et al. 2010; Donofrio et al. 2011; Lynch et al. 2014).

A recent study (Panigrahy et al. 2015) has shown that neurocognitive deficits in adolescents with dextro‐transposition of the great arteries (d‐TGA) repaired in early infancy are driven by differences in global white matter structural network topology, as determined via diffusion tensor imaging (DTI), graph analysis, and statistical mediation models. DTI enables investigation of white matter microstructure as well as connectivity between gray matter regions. Graph analysis involves a systems‐level approach to modeling the brain (the “connectome”) and the subsequent investigation of brain network topology. Differences in network topology have been seen in a variety of neurobehavioral disorders including schizophrenia and autism (Li et al. 2014; Griffa et al. 2015). Mediation models are useful for investigation of what brain differences underlie neurocognitive or neurobehavioral differences (Hayes 2009). Topological structural differences may constitute powerful mediators of neurocognitive outcome and may represent potent biomarkers for not only neurocognitive outcome but also for therapy to optimize it. Since children with CHD have greater likelihood of ADHD (Shillingford et al. 2008), we hypothesized that differences in brain structural topology may underlie this diagnosis as well. The risk for ADHD children with complex CHD may, in fact, be 3–4 times higher as in the general population (Shillingford et al. 2008), making it important to understand the neurophysiological etiology.

As an exploratory analysis, we wished to investigate whether different alleles of apolipoprotein E (*APOE*) interact with d‐TGA and modulate ADHD; this interaction may also be mediated via changes in brain network topology. ApoE‐containing lipoproteins are the primary lipid transport vehicles in the CNS and have an important role in mobilization and redistribution of cholesterol and phospholipids during remodeling of neuronal membranes (Laskowitz et al. 1998). We previously identified and validated an association of *APOE* genotype with early neurodevelopmental outcomes after cardiac surgery in neonates and infants with CHD (Gaynor et al. 2003, 2009, 2014). The *APOE* ε2 allele (described in Materials and Methods) was associated with worse performance at 12–14 months of age after surgery and with an increased risk of behavior problems at 4 years of age, while the *APOE* ε4 allele was associated with better outcomes.

Thus, we used graph analysis techniques on DTI data acquired from adolescents with d‐TGA corrected in early infancy and prospectively enrolled in the Boston Circulatory Arrest Study (BCAS). Our metrics, which reflect network connectivity, segregation, integration, and the balance between integration and segregation, were included in statistical mediation models with d‐TGA status or *APOE* genotype as the independent variable and ADHD outcome as the dependent variable.

## Materials and Methods

Details of the study population, *APOE* genotyping, the MRI acquisition details, the graph analysis technique, and statistical mediation models are available from previously published work (Bellinger et al. 1995, 2011; Tardiff et al. 1997; Gaynor et al. 2003; Rivkin et al. 2013; Rollins et al. 2014; Panigrahy et al. 2015) and are only summarized here.

### Participants

Study participants with d‐TGA were taken from the BCAS; healthy referent adolescents met standard criteria from the NIH MRI study of normal brain development. This study was approved by the Boston Children's Hospital Institutional Review Board and adhered to both institutional guidelines and the Declaration of Helsinki. Parents provided written informed consent, and adolescents provided assent.

### *APOE* Genotyping

*APOE* genotype was determined according to single‐nucleotide polymorphisms that specify a cysteine and/or an arginine residue at codons 112 and 158 of the *APOE* gene. The ε2 allele consists of a cysteine at positions 112 and 158; ε3 consists of a cysteine at position 112 and an arginine at position 158; ε4 consists of an arginine at both positions.

### DTI acquisitions and preprocessing

**Figure 1:**
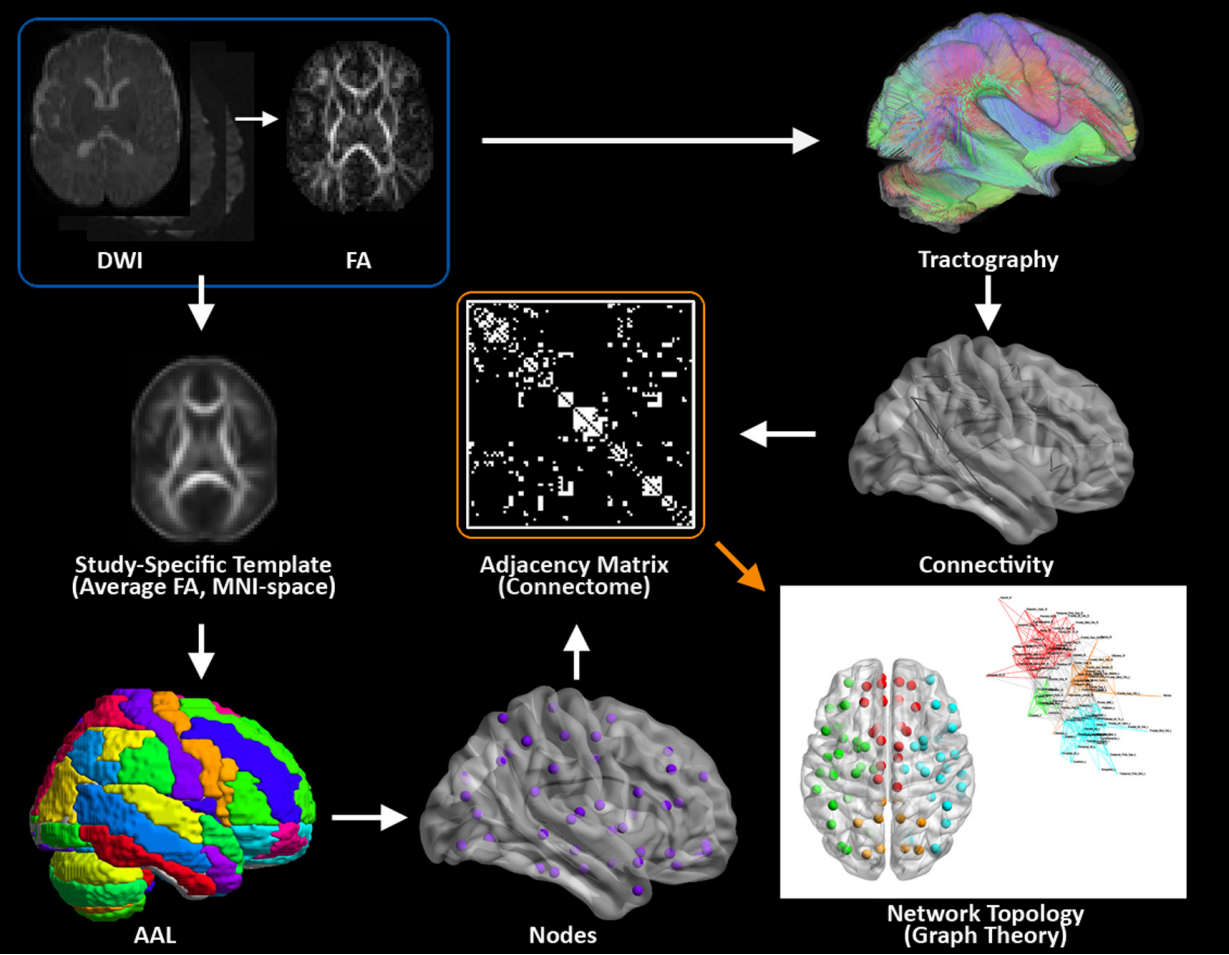
Methodology for Structural Network Topology Analysis. A flow diagram for the construction of WM structural networks from DTI including registration, segmentation, generation of WM fiber tracts using deterministic tractography, and generation of adjacency matrix and nodes (see Panigrahy et al. 2015 for more details).

DTI data were obtained from a GE Twin 1.5T system (General Electric, Milwaukee, WI) at Boston Children's Hospital with *b* = 750 s/mm^2^ and six diffusion‐encoding directions. Standard preprocessing was used with tools in FSL (FMRID, RRID: SCR_002823, http://www.fmrib.ox.ac.uk/fsl/). Deterministic tractography was performed using in‐house software written in IDL (http://www.ittvis.com, Boulder, CO). DTI data were segmented into 76 anatomical regions by applying the automated anatomic labeling (AAL) template (Tzourio‐Mazoyer et al. 2002) transformed into native space (Fig. 1). Adjacency matrices (unweighted graphs) were computed, with each off‐diagonal element containing 1 if at least one streamline connected the two regions, or 0 if not.

### Graph analysis

Graph metrics (global efficiency, modularity, transitivity, and small‐worldness) (Rubinov and Sporns 2010) were computed via the C*++* modules available from the Brain Connectivity Toolbox (BCT; RRID:SCR_004841, http://www.brain-connectivity-toolbox.net). A brief summary of each graph metric is provided here; we refer the reader to (Rubinov and Sporns 2010) for a more detailed description.

#### Cost

The ratio of connections in the graph to the total possible number of connections.

#### Global efficiency

For each pair of nodes, the efficiency is the reciprocal of the shortest path length between those nodes (if the nodes are disconnected, the efficiency is zero). Global efficiency is the efficiency averaged over all pairs of nodes.

#### Modularity

The graph is segregated into subnetworks and the modularity measures the connections within subnetworks as compared to the connections between subnetworks. The Louvain algorithm (Blondel et al. 2008) is used to optimize the subnetwork assignments.

#### Transitivity

Transitivity is the ratio of the actual number of “triangles” in the graph to the possible number of “triangles” (e.g., if node A is connected to node B, and node A is connected to node C, is node B connected to node C).

#### Small‐worldness

A “small‐world” network has similar global efficiency, but much greater transitivity, when compared to a random graph with the same degree distribution. The small‐worldness parameter is defined as the ratio of global efficiency in the graph to the global efficiency of a random graph, multiplied by the ratio of transitivity in the graph to the transitivity of a random graph.

### Neuropsychological testing

Neuropsychological test scores of adolescents with d‐TGA and healthy referent adolescents were reported previously in detail (Bellinger et al. 2011). A previous publication (Panigrahy et al. 2015) focused on relations between d‐TGA, structural network topology, and neurocognitive outcomes (e.g., WISC‐IQ, WIAT scores, memory tests, etc.) Here we focus on inattentiveness and hyperactivity. We used the Connors ADHD/DSM‐IV Scales (CADS) questionnaire administered to parents, teachers, and subjects. This questionnaire yields three scores linked to DSM‐IV criteria for ADHD: Hyperactive/Impulsive, Inattentive, and Combined. In addition, it yields an ADHD Index score based on the 12 items of the questionnaire that best distinguish children who have a diagnosis of ADHD from children who do not. Thus, 12 scores were obtained for each subject (4 scores obtained from parent, teacher, and subject each) in which higher score indicates worse performance.

### Mediation analysis

Statistical mediation models incorporated d‐TGA status or *APOE* genotype as the independent variable, graph metrics as the mediating variable, and (each of the 12) CADS scores as the dependent variable. When *APOE* genotype was the independent variable, comparisons were performed between d‐TGA subjects homozygous for the ε3 allele and those heterozygous for either the ε2 or ε4 allele and the ε3 allele. Additionally, the total effect (which is a simple linear regression model with d‐TGA status or *APOE* allele the independent variable and CADS score the dependent variable) was computed for d‐TGA and APOE allele. For all analyses, age, sex, and square root of acquired DTI frames were included as covariates. Bootstrapping (25,000 iterations) was used to test the mediation results for statistical significance. Results were deemed significant at a False Discovery Rate (FDR) corrected *q* < 0.05 (corrected for multiple comparisons across the 12 scores obtained from the CADS).

**Figure 2:**
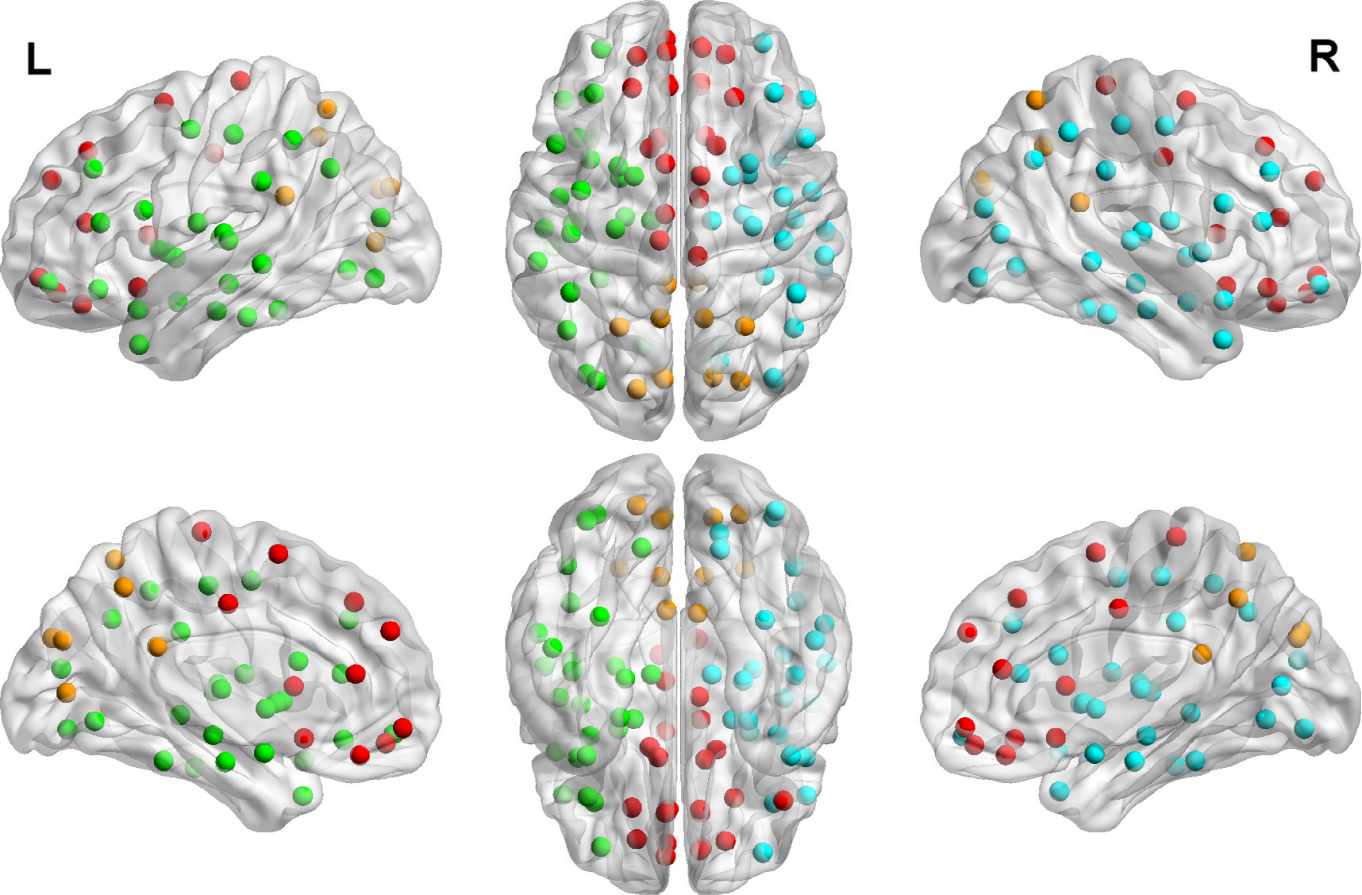
Subnetworks found from graph analysis. Performing graph analysis (modularity) on the participant‐wise averaged adjacency matrices (thresholded at cost = 0.15) yielded four subnetworks: left and right intrahemispheric (green and blue, respectively); and frontal and posterior interhemispheric (red and brown, respectively).

In addition to investigating global structural network topology, we performed regional (subnetwork) analyses. The graphs were averaged over all participants and, using the Louvain modularity algorithm (Blondel et al. 2008), regional modules (subnetworks) were found consisting of frontal interhemispheric, posterior interhemispheric, left intrahemispheric, and right intrahemispheric regions (Fig. 2). Using BCT, nodal metrics (degree, nodal efficiency, clustering coefficient, participation coefficient) were computed and averaged over all nodes in each subnetwork. Degree was further separated into intramodular degree (i.e., connections to nodes within the same subnetwork) and intermodular degree (i.e., connections to nodes in different subnetworks). (We again refer the reader to (Rubinov and Sporns 2010) for a detailed description of these metrics.)

## Results

### Clinical trial participants

Data from 47 adolescents with d‐TGA and 29 referent subjects were included in the final analysis (Rivkin et al. 2013). The scans of an additional 33 adolescents with d‐TGA and 11 referent subjects were excluded from analysis due to unacceptable signal artifact. Demographic characteristics (gestational age, sex, and age at MRI) in the excluded subjects did not differ from those of subjects included in the final analysis. Adolescents with d‐TGA, compared to referent subjects, were older at MRI, more likely to be male, and lower in social class, but had similar gestational age. Of the d‐TGA patients, 31 were homozygotes for the ε3 allele, five were ε3–ε4 heterozygotes, nine were ε2–ε3 heterozygotes, and two were ε2–ε4 heterozygotes (these two were not included in the APOE genotype analysis).

### Analysis of total effect

Subjects with d‐TGA, compared to referent subjects, demonstrated worse scores (FDR corrected *q* < 0.05) on two CADS tests (Parent report: combined, inattentive); significance was not reached for the other 10 tests. Significance was not reached for d‐TGA subjects with the *APOE* ε2 allele or the ε4 allele on any test.

### Analysis of indirect effect

In the following sections, we present significant results from the mediation analyses (analyses of indirect effect). We remind the reader that an indirect effect (e.g., better/worse CADS scores in d‐TGA adolescents mediated by differences in a network topology metric) does not necessarily imply a significant total effect. In addition to the specific metric, we list the specific CADS tests for which significance was reached (Adolescent, parent, or teacher report: and then the specific subtest(s), whether combined, hyperactive/impulsive, total, or inattentive).

### Neurobehavioral mediation analysis: effect of d‐TGA

**Figure 3:**
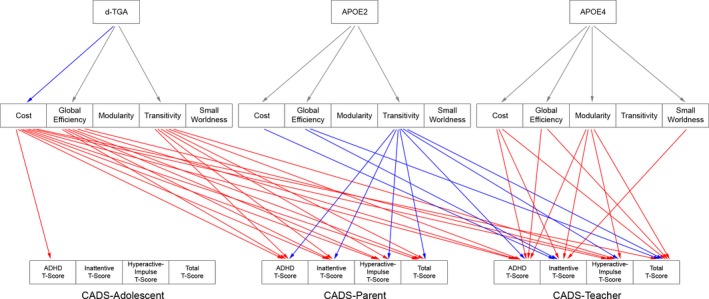
Global Structural Network Topology Mediates CADS Scores in d‐TGA Adolescents with and without the ε4 and ε2 ApoE alleles. Results from the mediation analyses demonstrate different paths by which global topology differences mediate CADS scores for d‐TGA adolescents in general (d‐TGA), and for d‐TGA adolescents with the ε2 or ε4 ApoE alleles (APOE2; APOE4). Arrows from the topology metrics to the CADS scores indicate a significant indirect effect (FDR corrected for multiple comparisons with *q* < 0.05; red = positive, blue = negative). Arrows from the independent variables to the topology metrics indicate difference in topology metrics (red = positive, blue = negative, gray = difference not significant).

At the global level, worse (higher) CADS scores (Fig. 3) in d‐TGA adolescents were mediated (FDR corrected *q* < 0.05) by differences in: *cost* (Adolescent report: combined; Parent report: all tests; Teacher report: combined, hyperactive/impulsive), *global efficiency* (Parent report: inattentive, hyperactive/impulsive, total; Teacher report: combined, hyperactive/impulsive), and *transitivity* (Parent report: all tests; Teacher report: combined, hyperactive/impulsive). Cost, efficiency, and transitivity reflect overall connectivity, integration, and segregation, respectively.

**Figure 4:**
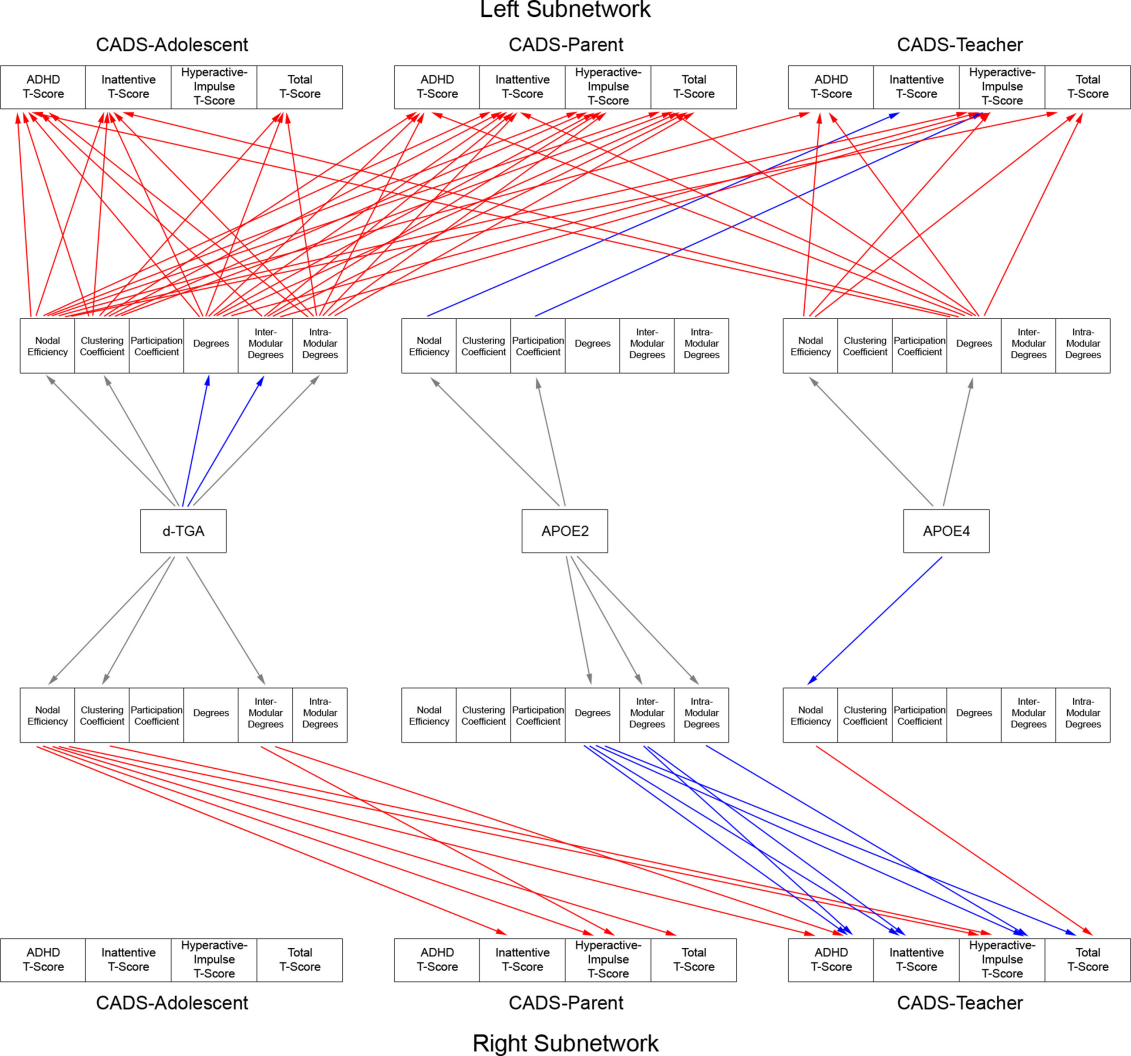
Intrahemispheric Regional Structural Network Topology Mediates CADS Scores in d‐TGA Adolescents with and without the ε4 and ε2 ApoE alleles. Results from the mediation analyses demonstrate different paths by which regional topology differences (in the left and right intrahemispheric networks) mediate CADS scores for d‐TGA adolescents in general, and for d‐TGA adolescents with the ε2 or ε4 ApoE alleles. Arrows from the topology metrics to the CADS scores indicate a significant indirect effect (FDR corrected for multiple comparisons with *q* < 0.05; red = positive, blue = negative). Arrows from the independent variables to the topology metrics indicate difference in topology metrics (red = positive, blue = negative, gray = difference not significant).

At the regional level, indirect effects (FDR *q* < 0.05) were mainly seen in the left and right intrahemispheric subnetworks (Fig. 4). In the left intrahemispheric subnetwork, worse scores were mediated by: degree (Adolescent report: combined, inattentive, total; Parent report: all subtests; Teacher report: hyperactive/impulsive), intermodular degree (Adolescent report: combined; Parent report: inattentive, hyperactive/impulsive, total; Teacher report: hyperactive/impulsive), intramodular degree (Adolescent report: combined, inattentive, total; Parent report: all subtests), nodal efficiency (Adolescent report: combined, inattentive, total; Parent report: inattentive, hyperactive/impulsive; Teacher report, combined, hyperactive/impulse, total), and clustering coefficient (Adolescent report: combined, inattentive, total; Parent report: all tests). Degree, nodal efficiency, and clustering coefficient are reflective of connectivity, integration, and segregation, respectively, at the subnetwork level. In the right intrahemispheric network, worse scores were mediated by: intermodular degree (Parent report: hyperactive/impulsive; Teacher report: combined), clustering coefficient (Teacher report: hyperactive/impulsive), and nodal efficiency (Parent report: all subtests; Teacher report: combined, hyperactive/impulsive).

**Figure 5:**
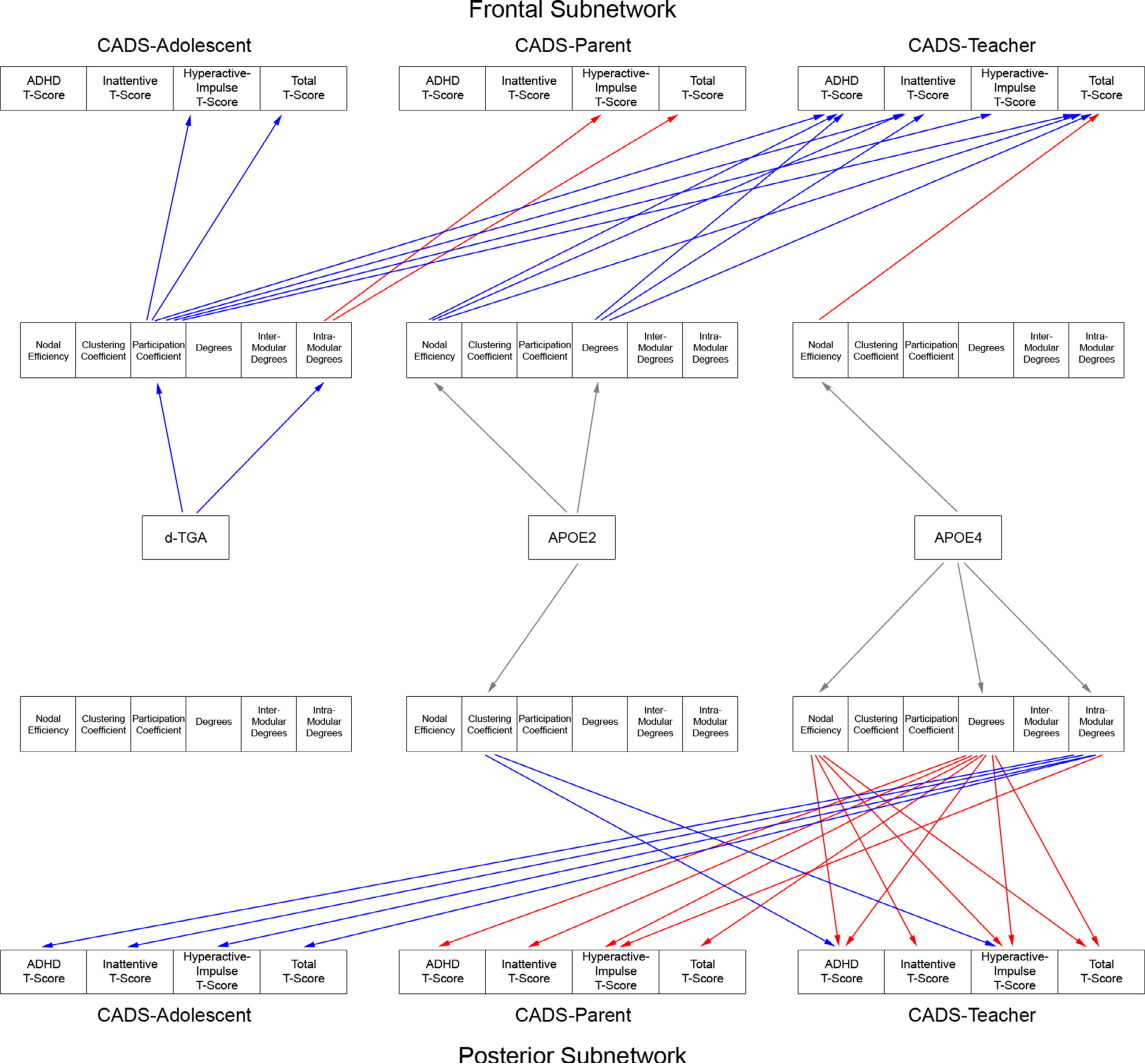
Interhemispheric Regional Structural Network Topology Mediates CADS Scores in d‐TGA Adolescents with and without the ε4 and ε2 ApoE alleles. Results from the mediation analyses demonstrate different paths by which regional topology differences (in the frontal and posterior interhemispheric networks) mediate CADS scores for d‐TGA adolescents in general, and for d‐TGA adolescents with the ε2 or ε4 ApoE alleles. Arrows from the topology metrics to the CADS scores indicate a significant indirect effect (FDR corrected for multiple comparisons with *q* < 0.05; red = positive, blue = negative). Arrows from the independent variables to the topology metrics indicate difference in topology metrics (red = positive, blue = negative, gray = difference not significant).

Significant indirect effects (FDR *q* < 0.05) were also seen in the frontal interhemispheric network (Fig. 5). Worse scores were modulated by intramodular degree (Parent report: hyperactive/impulsive, total). Interestingly, better (lower) scores were mediated by participation coefficient (Adolescent report: hyperactive/impulsive, total; Teacher report: all subtests). Participation coefficient (in addition to clustering coefficient) also reflects network segregation.

### Neurobehavioral mediation analysis: effect of APOE ε4 genotype

Our exploratory aim was to determine whether the differences in network topology mediated differences in ADHD functioning in d‐TGA adolescents heterozygous for either the ε2 or the ε4 allele as compared to those homozygous for the ε3 allele. At the global level (Fig. 3), worse scores in d‐TGA adolescents with the ε4 allele were mediated by differences in: cost (Teacher report: combined, inattentive, total), global efficiency (Teacher report: combined, total), modularity (Teacher report: all tests), and small‐worldness (Teacher report: inattentive). (Small‐worldness is a metric representing the balance between integration and segregation.)

At the regional level, worse scores were mediated (FDR *q* < 0.05) by topology differences in all subnetworks (Figs. 4, 5). Right intrahemispheric: worse scores were mediated by nodal efficiency (Teacher report: total). Left intrahemispheric: worse scores were mediated by nodal efficiency (Teacher report: combined, hyperactive/impulsive, total), and degrees (Adolescent report: combined, inattentive; Parent report: combined, inattentive, total; Teacher report: combined, total). Posterior interhemispheric: worse scores were mediated by nodal efficiency (Teacher report: all subtests), degrees (Parent report: all subtests; Teacher report: combined, hyperactive/impulsive, total), and intramodular degree (Parent report: hyperactive/impulse). Frontal interhemispheric: worse scores were mediated by nodal efficiency (Teacher report: total). Interestingly, in the posterior interhemispheric subnetwork, better scores were also mediated by intramodular degree (Adolescent report: all tests).

### Neurobehavioral mediation analysis: effect of APOE ε2 genotype

At the global level (Fig. 3), better (lower) CADS scores in d‐TGA adolescents with the ε2 allele were mediated (FDR *q* < 0.05) by differences in: cost (Teacher report: inattentive), global efficiency (Teacher report: inattentive, total), and transitivity (Parent report: all tests; Teacher report: combined, hyperactive/impulsive, total).

Regionally, better (lower) CADS scores were mediated (FDR *q* < 0.05) by topology differences in all subnetworks (Figs. 4, 5). Frontal interhemispheric: better scores were mediated by nodal efficiency (Teacher report: combined, inattentive, total), and degrees (Teacher report: combined, inattentive, total). Posterior interhemispheric: better scores were mediated by clustering coefficient (Teacher report: combined, hyperactive/impulsive). Right intrahemispheric: better scores were mediated by degree (Teacher report: all subtests), intermodular degree (Teacher report: combined, inattentive), intramodular degree (Teacher report: hyperactive/impulsive). Left intrahemispheric: better scores were mediated by nodal efficiency (Teacher report: inattentive) and participation coefficient (Teacher report: hyperactive/impulsive).

## Discussion

Previous work has demonstrated that diminished cognitive function (including overall intelligence, memory, and executive function) in adolescents with d‐TGA relates to white matter microstructure (Rollins et al. 2014) and is mediated by global differences in white matter structural network topology (Panigrahy et al. 2015). These findings suggest that alteration of large‐scale network organization can adversely affect cognitive dysfunction in children with surgically treated CHD. We now report that scores on CADS ADHD tests are also mediated by white matter global and regional topologic differences. Additionally, our exploratory analysis found that *APOE* genotype exerted an effect on structural topology and CADS scores in these adolescents.

We found worse scores on two CADS subtests (Parent report: combined, inattentive) in the subset of d‐TGA adolescents for which DTI data were acquired, in agreement with DeMaso et al. (2014) and Bellinger et al. (2011) who earlier showed worse scores in Parent‐report CADS index scores in the entire BCAS cohort. Our results are also in agreement with Shillingford et al. (2008) who demonstrated higher prevalence of ADHD in school‐aged (5–10 years old) children with CHD. Interestingly, as was the case in our study, this higher prevalence was more pronounced using the parent‐report scores as compared with the teacher‐report scores. The reason for this is unknown, but may be related to teachers having a higher threshold for classifying a behavior as abnormal. Taken together, these results indicate that vulnerability for ADHD is present in the CHD cohort throughout childhood and adolescence.

However, statistical significance was not reached for the analyses regarding APOE genotype. While this may be due to insufficient power, an effect of *APOE* genotype on ADHD outcome in general has not been found (see (Gatt et al. 2015) for meta‐analyses), although these studies were conducted on healthy referents and may not be transferable to CHD populations. A previous study (Gaynor et al. 2009) also did not find a significant effect regarding ADHD specifically. However, this study was conducted using children of preschool age who were much younger than those of this study.

We also found that the worse ADHD outcomes in d‐TGA adolescents are mediated by global structural topology differences (cost, global efficiency, and transitivity). The topology differences involving network segregation (transitivity) differed, however, from those (modularity and small‐worldness) found to mediate poorer neurocognitive outcomes (Panigrahy et al. 2015). This suggests that different structural features may underlie cognitive as compared to behavioral deficits such as inattentiveness and hyperactivity in d‐TGA subjects.

Our preliminary evidence here also suggests an influence of *APOE* genotype on structural topology and ADHD outcome. Better ADHD outcome in d‐TGA adolescents with the ε2 allele was mediated by differences in global network topology (cost, global efficiency, and transitivity). By contrast, in d‐TGA adolescents with the ε4 allele, worse ADHD outcome was mediated by a different set of global metrics (cost, global efficiency, modularity, and small‐worldness). Interestingly, these results in adolescents are opposite to what was found (Gaynor et al. 2009) in CHD patients of preschool age (i.e., the ε2 allele is deleterious while the ε4 allele is neuroprotective).

Our results indicate that network integration, as reflected by global efficiency, mediates a variety of neurobehavioral outcomes, including the worse ADHD outcomes seen in this study as well as worse neurocognitive function seen in our previous report (Panigrahy et al. 2015). With regard to network segregation, however, transitivity was found to mediate worse ADHD outcome, in contrast to small‐worldness and modularity mediating worse neurocognitive outcome. Modularity is a measure representing network segregation at the regional (i.e., subnetwork) level; a more modular network has a greater proportion of intra‐subnetwork connections. Transitivity, on the other hand, represents network segregation at the nodal level and represents the proportion of “triangles” (e.g., if node A is connected to node B and node B is connected to node C, node A is also connected to node C). Small‐worldness is related to the ratio of transitivity to global efficiency and is often interpreted as the “balance” between network integration and segregation (Watts and Strogatz 1998). Thus, the increased modularity underlying poorer neurocognitive performance may indicate a deficit in long‐range connectivity between subnetworks, as efficient communication between subnetworks is necessary for complex cognitive tasks (Li et al. 2009). By contrast, the altered transitivity associated with worse ADHD may be more associated with short‐range and local connectivity. This hypothesis is supported by DTI studies (Davenport et al. 2010; van Ewijk et al. 2014) showing uniquely higher FA in ADHD adolescents (compared to adolescents with schizophrenia) in the left and right anterior corona radiata, and higher FA in widespread white matter regions associated with short‐range connectivity.

Our results from our exploratory analysis also suggest an important role of the ApoE genotype in brain development and recovery after injury. However, the genotype effect identified in this study (the ε2 allele associated with better outcomes and the ε4 allele with worse outcomes) is opposite to that seen in previous studies involving younger age ranges (Gaynor et al. 2003, 2009). Thus, our results suggest antagonistic pleiotropy, which occurs when genes control for some beneficial traits and some deleterious traits. An example is, when a gene is associated with a beneficial effect early in development, thereby enhancing selection and survival, but has a more deleterious effect later in life, resulting in the development of disease (Han and Bondi 2008; Rusted et al. 2013). In the normal population, the ε4 allele is neuroprotective early in development, and has been associated with a decreased risk of spontaneous abortion (Zetterberg et al. 2002) and improved neurodevelopmental outcomes in infants with malnutrition (Oria et al. 2007, 2010) or lead exposure (Wright et al. 2003). Thus, improved early neurodevelopmental outcomes after cardiac surgery may be the result of greater neuroprotection against the effects of hypoxic‐ischemic injury occurring during the third trimester or perinatally. However, the ε4 allele may interact in a deleterious manner with other risk factors later in development, in a similar manner as seen in normal adults regarding increased risk for AD and worse outcome after TBI (Shu et al. 2014; Tsao et al. 2014). The exact timing of this switchover remains unclear as do its environmental and genetic determinants. Similarly, the ε2 allele, while interacting deleteriously with injury caused by CHD pre‐ or perinatally, may provide an advantage later in development. Interestingly, the same metrics (cost, global efficiency, and transitivity) which mediate better ADHD outcomes for d‐TGA adolescents with the ε2 allele mediate worse ADHD outcomes in d‐TGA adolescents in general; we speculate that the ε2 allele counteracts the general effect of d‐TGA on the developing brain. Further research will be necessary to investigate this hypothesis further.

In this study, we went beyond global metrics to also look at regional topology differences. The changes in topology mediating worse CADS scores in d‐TGA adolescents were seen to originate predominantly from the left and right intrahemispheric networks (primarily the left), and in the nodal metrics (degree, nodal efficiency, clustering coefficient) corresponding to those previously seen at the global level (cost, global efficiency, transitivity). In the frontal interhemispheric network, however, while intramodular degree mediated worse CADS scores, participation coefficient mediated better scores. Intramodular and intermodular degrees were significantly lower in d‐TGA patients, while participation coefficient was reduced at a trend level. Based on these results, we hypothesize that d‐TGA results in impaired frontal‐frontal (including interhemispheric) connectivity adversely affects CADS scores. However, a partial compensatory mechanism is available via reduced connectivity to other regions of the brain. Interestingly, the previously cited study (van Ewijk et al. 2014) showed decreased FA in frontal regions in children with a diagnosis of ADHD, but a positive correlation of FA with ADHD symptoms in those children. The decreased overall FA may correspond to the impaired frontal‐frontal connectivity found here, while the positive correlation of FA with ADHD symptoms may correspond to the reduced connectivity to the other regions, resulting in less crossing fibers.

Regarding the ε2 and ε4 alleles, changes in regional topology mediating better ADHD outcomes were seen throughout the brain, indicating that the effects of these alleles may not be regionally specific. Interestingly, topology metrics in the posterior interhemispheric networks for the ε4 allele mediated worse scores in parent and teacher‐report scores but better scores for the self‐report scores. The correlation between parent, teacher, and adolescent CADS scores is quite weak overall (Kaner 2011), though with better correlations between parent teacher‐report scores, and the reliability of ADHD adolescent self‐reports for negative behaviors is questionable (Smith et al. 2000). Thus, we find it premature to speculate on the possible significance of these findings.

Our results regarding the effect of the ε2 or ε4 allele should be taken as preliminary due to the small number of participants heterozygous for each allele, and the consequent failure to control for clinical and perioperative variables. As a result of the small sample size, statistical significance for the indirect effect was sometimes reached in the absence of statistical significance for the total effect or either of the two individual pathways. Nevertheless, the preliminary evidence for antagonistic pleiotropy in these alleles is quite intriguing and awaits replication in a study with a larger sample size.

Additional limitations include use of a DTI sequence with six diffusion‐encoding gradient directions, the minimum necessary to compute the diffusion tensor. While greater SNR is available when more directions are used (Jones 2004), six directions provide comparable robustness for computation of parameters from deterministic tractography (Lebel et al. 2012). This method has been used to show differences in graph metrics (Shu et al. 2011) in patients with multiple sclerosis. However, there may exist even more relevant differences in brain network topology than were detected in our study. Future research may incorporate diffusion spectrum imaging (DSI) or Q‐ball imaging, which use a much larger number of directions.

## Conclusion

White matter structural network topology (including integration and segregation) is shown to mediate worse ADHD outcomes in adolescents with d‐TGA. The segregation metric (transitivity) is distinct from metrics of segregation (modularity, small‐worldness) previously found to mediate worse neurocognitive performance. Opposite to what was observed in CHD patients at younger ages, in this cohort, our exploratory analysis revealed that better outcome was mediated by structural topology in adolescents with the *APOE* ε2 allele while worse outcome was mediated in adolescents with the *APOE* ε4 allele. These results suggest an early switchover for the effects of antagonistic pleiotropy in individuals with complex CHD.

## Conflict of Interest

There has been no identified conflict of interest.
